# Schiff base complexes of copper and zinc as potential anti-colitic compounds

**DOI:** 10.1007/s10534-017-0016-z

**Published:** 2017-04-19

**Authors:** Elaine M. Conner, John Reglinski, W. Ewen Smith, I. Jack Zeitlin

**Affiliations:** 1 Department of Pure & Applied Chemistry, Strathclyde University, 295 Cathedral St., Glasgow, G1 1XL UK; 2 Department of Physiology and Pharmacology, Strathclyde University, 204 George St., Glasgow, G1 1XW UK

**Keywords:** Ulcerative colitis, Polymodal drugs, Sulfasalazine, Metal Schiff base compounds, Formalin induced colonic inflammation, TNB induced colonic inflammation

## Abstract

The design, synthesis and activity of polymodal compounds for the treatment of inflammatory bowel disease are reported. The compounds, being based on a metal-Schiff base motif, are designed to degrade during intestinal transit to release the bioactive components in the gut. The compounds have been developed sequential with the biomodal compounds combining copper or zinc with a salicylaldehyde adduct. These compounds were tested in a formalin induced colonic inflammation model in BK:A mice. From these studies a trimodal compound based on a zinc Schiff base analogue of sulfasalazine was designed. This was tested against a trinitrobenzenesulfonic acid (TNB) induced colitic model in Wistar rats. The use of two models allows us to test our compounds in both an acute and a chronic model. The trimodal compound reported is observed to provide anticolitic properties in the chronic TNB induced colitis model commensurate with that of SASP. However, the design of trimodal compound still has the capacity for further development. This the platform reported may offer a route into compounds which can markedly outperform the anti-colitic properties of SASP.

## Introduction

The two most important chronic inflammatory bowel diseases (IBD) are ulcerative colitis and Crohns colitis, with ulcerative colitis accounting for 65–85% of the total incidence of IBD (Shivananda et al. 1996; Burisch and Munkholm 2015). Both forms of colitis have an uncertain aetiology but have many features in common. Both are debilitating chronic diseases subject to exacerbations and remission, and both involve an immune component. The commonest symptoms are bloody diarrhoea, rectal bleeding, anorexia and weight loss. The colon shows extensive ulceration and there may be abscess formation (Kirsner and Shorter 1982). A severe attack can cause pathological colonic distension known as toxic dilation, or ulcer perforation resulting in faecal release into the abdomen. Both conditions are associated with high mortality (Sonnenberg 1990; Kassam et al. 2014).

**Fig. 1:**
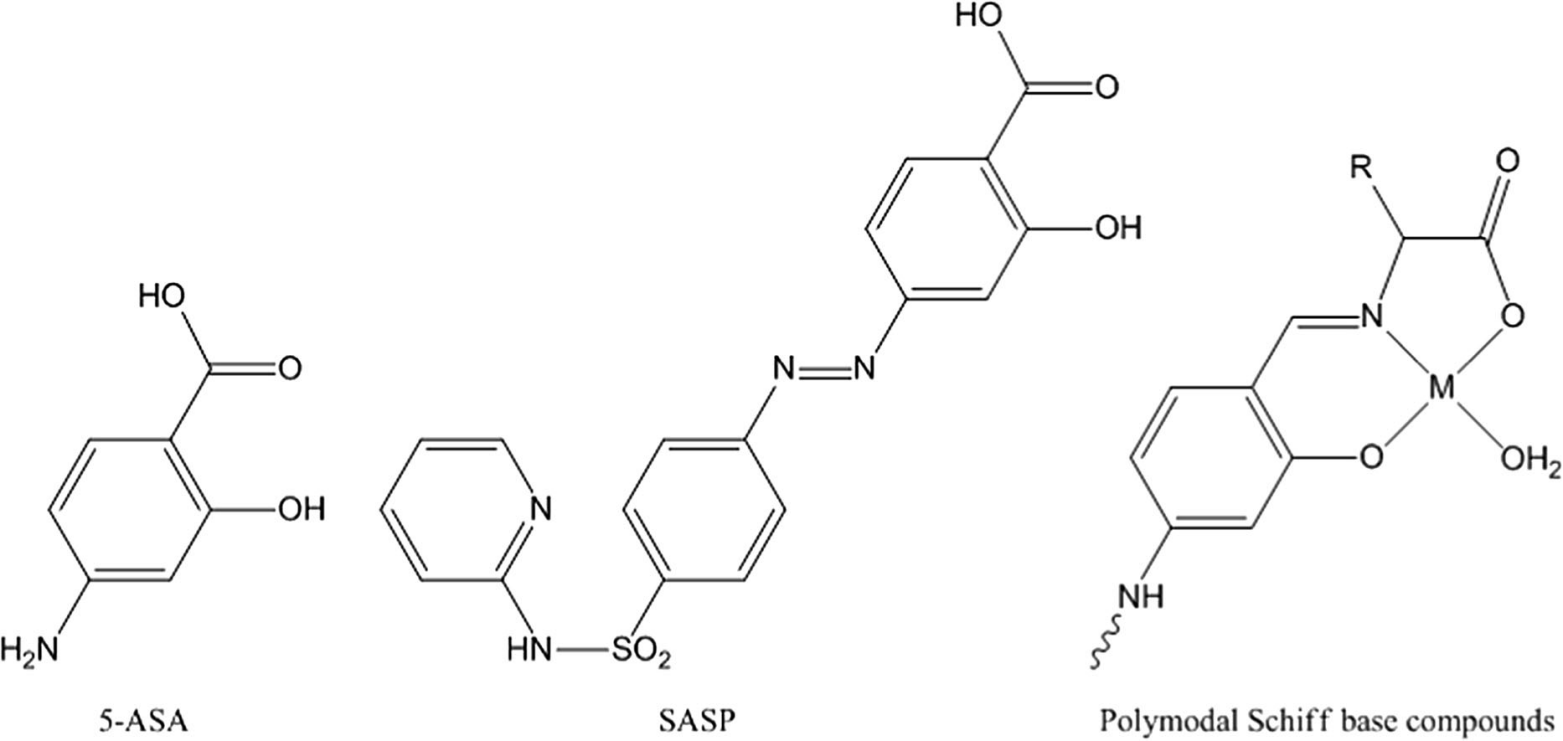
The structures of 5-ASA, SASP and the polymodal Schiff base complexes under study here for their anti-colitic properties. These Schiff base moieties potentially combine the activities of 5-ASA (in its reduced form) with a biologically relevant metal (M = Cu, Zn). The hydrolytically sensitive Schiff base linkage is generated via an interaction with a third entity (a simple amino acid in this study). The use of the reduced form of salicylic acid to form the Schiff base linkage allows the aniline residue of 5-ASA to be modified further and in line with SASP

There are few drugs available to treat IBD. The currently used non-steroidal anti-colitic compounds are all pro-drug formulations for 5-aminosalicylic acid (5-ASA, Fig. 1), which has to be delivered to the colon for its therapeutic effect (Friend 1991; Podolsky 2002; Furfaro et al. 2015). Orally administered 5-ASA is unstable in the acid environment of the stomach, and is also readily absorbed into the gastrointestinal tract before reaching the colon. The oldest of the 5-ASA prodrugs and still the main stay of maintenance therapy for colitis, is salicylazosulfapyridine (sulfasalazine, SASP, Fig. 1). This was developed by Svartz as an anti-rheumatic drug and found by chance to have a beneficial effect on colitis patients (Svartz 1942). When taken by mouth, 75–90% of the SASP reaches the colon intact where bacterial action cleaves the azo-bond to release 5-ASA. However, the sulfapyridine carrier is also absorbed through the colon wall and can produce toxic side effects as a result.

Attempts have been made to develop new formulations in an attempt to deliver 5-ASA to the colon using less toxic carriers (Friend 1991). Thus far none of these formulations is more active than SASP. Treatment with all of these drugs is frequently discontinued due to their limited effectiveness and because patients may show hypersensitivity reactions and other side effects. Currently, all that remains to such patients is treatment with cortico-steroids or immuno-suppressants such as azathipine, both approaches being accompanied by adverse systemic side effects. Until new chemical entities or biologics are identified (Danese et al. 2015; Hagan and Cross 2015), surgery will remain a common outcome if the attacks recur (Sorenson 1976; Milanino et al. 1985).

It is clear that new drugs are needed for controlling the symptoms and preventing the tissue damage associated with IBD. Metal complexes of active substances are useful lead compounds because certain metals such as copper and zinc have some biological activity and because as centres they can help protect the active drug in the stomach and assist its delivery to the colon (Sorenson 1976, 1977; Milanino et al. 1985; Dillon et al. 2004).

Copper complexes (e.g. copper salicylate) have been investigated as anti-arthritic and anti-ulcer agents and have demonstrated a positive benefit in a number of animal models of inflammation and inflammatory conditions (Sorenson 1976, 1977). In the absence of a detailed mechanism for their action a working hypothesis has been put forward which involves the formation of copper chelate complexes in the plasma that are responsible for the observed anti-inflammatory activity. However, it has also been suggested that the activation of copper dependant processes assists in wound and tissue repair (Borkow et al. 2008, 2010). Alternatively or additionally, the anti-inflammatory and anti-ulcer activity of copper complexes could be associated with the dismutation of the superoxide radical anion, which is routinely implicated in oxidative tissue damage. The therapeutic role of zinc has been studied less extensively than copper. Zinc deficiency has been related to poor would healing and zinc concentrations in peripheral blood cells are lower than normal in patients with inflammatory diseases such as rheumatoid arthritis (Svenson et al. 1985; Lansdown et al. 2007; Binneboesel et al. 2010; Hwang et al. 2012). Indeed zinc deficiency has been suggested to play a role in the formation and clinical course of fistulas in Crohns disease and a reason for poor would healing in inflammatory bowel disease (Kruis et al. 1985; Binneboesel et al. 2010; Hwang et al. 2012).

The search for new anti-colitic agents is hindered by the an incomplete picture of the aetiology of inflammatory bowel disease and the availability of representative animal models for the testing of such agents. Another factor to be considered is the targeting of the anti-colitic agent to the colon when oral administration is the preferred mode of delivery. There are two main approaches to colon specific drug delivery namely the use of metabolic events catalysed by the gut microflora or the use of enteric coatings (e.g. PENTASA®, Asacol) which have disintegration profiles based on the natural pH gradient along the gastrointestinal tract. Both aid the release of the active agents at the inflamed sites in the colon (Friend 1991). Localisation of 5-ASA release from these preparations is primarily dependant on transit time. Transit through the colon is considerably slower than through the small intestine and can range from 10 h to as long as 78 h (Wyman et al. 1978). However, transit times can be expected to be more rapid during an episode of the symptoms associated with IBD.

Parashar and co-workers have synthesised a series of Schiff base complexes derived from salicyaldehyde and 2-substituted analines (Parashar et al. 1989). The copper chelates did not show significant anti-inflammatory activity on carrageenan induced oedema and formaldehyde induced arthritis. Significantly, the copper complex of salicylidene and anthranilic acid was reported to possess anti-ulcer and anti-diarrhoeal activity (Sorenson 1976, 1977; Kruis et al. 1985). In contrast and more recently Alafeey et al. have reported on quinazolinone Schiff bases which have some anti-colitic activity (Alafeefy et al. 2015). Since it is possible to build these complexes from a series of active agents it is possible to quickly alter their biological effects and make them bi-and tri-modal such that they respond to the local gut microflora or local pH for activation. Furthermore the relative insolubility of metal Schiff base complexes may satisfy the requirements for new oral therapeutic agents for the treatment of IBD which have to survive transit through the digestive system. It has been demonstrated that metal complexes can be more potent than the parent ligands (Sorenson 1976, 1977). If this is true for Schiff base complexes then hydrolysis of the complex to its basic components in colon may not be essential to confer activity.

We aim to test the effectiveness of metal Schiff base complexes in controlling chronic inflammation. There is the potential for each part of the Schiff base molecule to comprise an anti-inflammatory agent with a different mode of action. This approach to combination therapy may be useful in conditions such as ulcerative colitis, which has a range of inflammatory mediators promoting the inflammatory process.

## Experimental

Unless otherwise stated the chemicals used were commercially obtained. Infra red spectra were recorded on a Perkin Elmer 457 grating infra-red spectrophotometer. ^1^H NMR spectra were recorded on a Bruker 250 MHz instrument. U.V. visible spectra were recorded on a Phillips PU8740 spectrometer.

### Preparation of zinc complexes

Salicylaldehyde (0.5 g, 4 mmol) and potassium hydroxide (0.25 g, 4 mmol) were mixed with an equimolar amount of the desired amino acid (4 mmol) in water. The bright yellow solution was heated at 40 °C. After 15 min a solution of zinc acetate (0.88 g, 4 mmol) in methanol (20 mL) was added and the cloudy mixture heated for a further 45 min at 60 °C. The solution was allowed to cool and the product collected, washed with ethanol and diethyl-ether. The product was then re-suspended in distilled water (150 mL) and stirred for 3 h. The remaining solid was recovered by filtration, washed with ethanol and diethyl-ether and dried *in*-*vacuo*.

#### Aqua-salicylidenealanine-zinc

Expected for C_12_H_9_NO_4_Zn.H_2_O: C, 41.05; H, 4.48; N, 4.79%: Found C, 41.05; H, 4.55; N, 4.70% ν (cm^−1^) 3400–3200 (OH); 1,635 (C=N); 1,595 (COO); 1,390 (CO). δ_H_ (250 MHz; solvent (CD_3_)_2_SO) 8.3 (s, 1H, CH=N), 7.1 (m, 2H, arom), 6.7 (d, 1H arom), 6.5 (t, 1H arom), 3.7 (q, 1H, CH), 1.3 (d, 3H, –CH_3_).

#### Aqua-salicylideneglycine-zinc

Expected for C_9_H_9_NO_4_Zn: C, 41.49, H, 3.48; N, 5.38%. Found C, 41.32; H, 3.23; N, 5.15%. ν (cm^−1^) 3350–3200 (OH); 1,630 (C=N); 1,595 (COO); 1,390 (CO). δ_H_ (250 MHz; solvent (CD_3_)_2_SO) 8.3 (s 1H CH=N), 7.1 (m, 2H, arom), 6.5 (d, 1H arom), 6.4 (t, 1H arom), 3.0 (d, 2H, –CH_2_–)

#### Aqua-salicylideneleucine-zinc

Expected for C_13_H_17_NO_4_Zn: C, 49.31; H, 5.41; N, 4.42% Found C, 48.81; H, 5.22; N, 4.33%. ν (cm^−1^) 3330–3100 (OH); 1,630 (C=N); 1,580 (COO); 1,390 (CO). δ_H_ (250 MHz; solvent (CD_3_)_2_SO) 8.2 (s 1H CH=N), 7.1 (m, 2H, arom), 6.5 (d, 1H arom), 6.4 (t, 1H arom), 3.6 (q, 1H, CH), 1.5 (br m, 3H, CH_2_ and CH), 0.9 (dd, 6H, –CH_3_).

### Preparation of copper complexes

Salicylaldehyde (0.5 g, 4 mmol) and potassium hydroxide (0.25 g 4 mmol) were mixed with an equimolar amount of the desired amino acid (4 mmol) in water (15 mL). The bright yellow solution was heated at 40 °C. After 15 min a solution of copper acetate (0.80 g, 4 mmol) in ethanol (20 mL) was added and the green mixture heated for a further 3 h at 60 °C. The solution was reduced by 80% and the solution left overnight to allow precipitation. The product was collected and washed with ethanol. The product was then re-suspended in the minimum amount of water filtered and re-precipitated with ethanol. The resulting solid was recovered by filtration, washed with ethanol and diethyl-ether and dried *in vacuo*.

#### Aqua-salicylidenealanine-copper

Expected for C_10_H_11_NO_4_Cu.H_2_O: C, 41.31; H, 4.51; N, 4.82%: Found C, 41.52; H, 3.80; N, 4.83% ν (cm^−1^) 3500–3200 (OH); 1,635 (C=N); 1,595 (COO); 1,390 (CO).

#### Bis-(aqua-salicylideneglycine-copper)

Expected for C_18_H_18_N_2_O_8_Cu_2_.H_2_O: C, 40.38; H, 3.76; N, 5.23%: Found C, 40.27; H, 3.66; N, 5.25% ν (cm^−1^) 3300–3100 (OH); 1,635 (C=N); 1,600 (COO); 1,395 (CO).

#### Aqua-salicylideneleucine-copper

Expected for C_13_H_17_NO_4_Cu: C, 49.59; H, 5.44; N, 4.45% Found C, 49.95; H, 5.39; N, 4.38%. ν (cm^−1^) 3330–3100 (OH); 1,630 (C=N); 1,600 (COO); 1,400 (CO).

### Preparation of 5-(p-sulfophenylazo)salicylidenealanine-zinc

Sulfanilic acid (8.65 g, 0.045 mol) and sodium carbonate (2.65 g, 0.025 mol) were dissolved in 50 mL of distilled water with heating (~60 °C). Once the solution stopped effervescing and became clear it was cooled to 15 °C. To this solution was added an aqueous solution of sodium nitrite (3.7 g, 0.0535 mol, 10 mL). The initial orange solution turned cloudy and white. This mixture was poured onto 60 g of crushed ice containing 10.5 mL of concentrated HCl. The diazotised sulfanilic acid was added to a cooled solution of salicylaldehyde (6 g, 0.05 mol in 60 mL of 3 M NaOH). The resulting yellow suspension was stirred for 3 h at 5 °C. The mixture was then heated to 80 °C and held there for 45 min. The solution was allowed to cool to room temperature whereupon an orange precipitate forms. This was collected washed with a small amount of cold water, ethanol and diethyl ether.

#### Sodium 5-(p-sulfophenylazo-)salicylaldehyde

Expected for C_13_H_9_N_2_O_5_SNa.2H_2_O: C, 42.88; H, 3.60; N, 7.69%: Found C, 43.57; H, 2.17; N, 7.41% ν (cm^−1^) 1,660 (C=O). δ_H_ (250 MHz; solvent (CD_3_OD) 9.9 (s 1H CHO), 8.1 (br s, 1H, arom), 7.95 (br d, 1H arom), 7.5 (d, 2H, arom–sulfanilic group), 7.0 (br d, 1H, arom), 6.8 (d, 2H, arom -sulfanilic group).

5-(p-sulfophenylazo-)salicylaldehyde (0.6 g, 2 mmol) dissolved in methanol (40 mL) was added to an ethanolic potassium hydroxide solution (1%, 25 mL). To this solution was added alanine (0.0.267 g, 3 mmol) dissolved in the minimum amount of water. The mixture was heated at 45 °C for 15 min. Zinc acetate (0.647 g, 3 mmol) dissolved in methanol (30 mL) was added and the mixture heated to 60 °C for 45 min. The volume of the solution was carefully reduced and an orange precipitate formed. This was collected, redissolved in methanol (with warming) and reprecipitated by trituration with di-ethyl ether.

#### Sodium 5-(p-sulfophenylazo-)salicylidenealanine-zinc

Expected for C_16_H_15_N_3_O_7_SNaZn.H_2_O: C, 38.46; H, 3.43; N, 8.41%: Found C, 38.06; H, 2.81; N, 8.05% ν (cm^−1^) 1,645 (C=N). δ_H_ (250 MHz; solvent (CD_3_OD) 8.4 (s 1H CH=N), 7.9 (m, 6H, arom), 7.0 (d, 1H, arom), 4.0 (q, 1H, CH), 1.5 (d, 3H, Me).

### pH stability studies

The desired metal complex (0.2 mmol) was dissolved in aliquots of dilute HCl (10 mL) which had been carefully adjusted to pH’s of 1, 2, 3, 4, 5, 7 respectively. The solutions were stirred for 3 h at 60 °C. The u.v.-visible spectrum (250–800 nm) was then recoded.

The desired metal complex (0.2 mmol) was dissolved in aliquots of dilute HCl (10 mL) which had been carefully adjusted to pH 1.5 or 2.5. Octanol (10 mL) was then added and the solutions stirred. At 2 min intervals the octanol layer was removed and its u.v.-visible spectrum (250–800 nm) recorded.

### Formalin induced colonic inflammation—protocols and validation (modified from Walsh et al. 1987)

#### Dosage

BK:A mice (25–30 g) received 0.2 mL of formalin (0.4, 0.8, 1.0, and 2.0%) intrarectally. Instillation was performed using a trocar needle inserted 2 cm proximal to the anus. One group received 0.2 mL of 0.9% saline intrarectally. Animals were sacrificed 3 days and 5 days later and the percentage tissue water content of the colonic tissue determined. Colons were examined for signs of inflammation using a scoring system. Six mice were used.

#### Time course

BK:A mice (25–30 g) received 0.2 mL of 0.8% formalin intrarectally. Instillation was performed using a trocar needle inserted 2 cm proximal to the anus. Test animals were killed at hourly intervals. Control animals, groups of six, received 0.2 mL of 0.9% saline intrarectally and were killed at the same time as the test animals. Time points studied were 0, 1, 2, 3, 4 and 5 h and the percentage tissue water content of the colonic tissue determined. Ten mice were used.

### The effect of intrarectal administration of 5-ASA, prednisolone, ZnSA, CuSA and zinc chloride on formalin induced colonic inflammation

#### 5-ASA

Male BK:A mice (20–25 g) in drug-treated groups received intrarectal administration of 0.2 mL of 5-ASA (15, 30, 150 mg kg^−1^) dissolved in 0.02 M sodium hydroxide using a trocar needle. Insertion was 2 cm proximal to the anus. A control group received 0.2 mL of 0.2 M sodium hydroxide intrarectally. Drug or vehicle was administered 3 days prior to, on the day of, and 1 day after formalin challenge. Colonic inflammation was induced via the local instillation of 0.2 mL of 2.0% formalin using a trocar needle inserted 2 cm proximal to the anus. A control group received 0.2 mL of 0.9% saline intrarectally. All animals were killed 2 days after formalin challenge. Colons were dissected out and examined for signs of inflammation using a graded scoring system (vide infra) and the percentage tissue water content calculated. Colonic tissue samples were frozen to assay for myeloperoxidase activity (vide infra). Groups of eight mice were used.

#### Prednisolone

Male BK:A mice (20–25 g) in drug-treated groups received i.p. injections (0.2 mL) of prednisolone (15, 30 mg kg^−1^ in 20% ethanol). One group received i.p. injections of 20% ethanol. Drug or vehicle was administered daily 3 days prior to on the day of and 1 day after formalin challenge. Colonic inflammation was induced via the local instillation of 0.2 mL of 2.0% formalin using a trocar needle. Insertion was 2 cm proximal to the anus. A control group received 0.2 mL of 0.9% saline intrarectally. All animals were killed 2 days after formalin challenge. Colons were dissected out and examined for signs of inflammation using a graded scoring system and the percentage tissue water content calculated. Colonic tissue samples were frozen to assay for myeloperoxidase activity. Groups of eight mice were used.

#### ZnSA/CuSA/ZnCl_2_

Male BK:A mice (20–25 g). in treatment groups received either ZnSA, CuSA or zinc chloride (1.0 and 5.0 mol kg^−1^) intrarectally using a trocar needle. ZnSA and CuSA were dissolved in 0.02 M sodium hydroxide (0.2 mL) whereas zinc chloride was prepared in distilled water (0.2 mL). Insertion was 2 cm proximal to the anus. A control group received 0.2 mL of 0.2 M sodium hydroxide intrarectally. Drug or vehicle was administered twice daily 3 days prior to, on the day of and 1 day after formalin challenge. Colonic inflammation was induced via the local instillation of 0.2 mL of 2.0% formalin using a trocar needle. Insertion was 2 cm proximal to the anus. A control group received 0.2 mL of 0.9% saline intrarectally. All animals were killed 2 days after formalin challenge. Colons were dissected and examined for signs of inflammation using a graded scoring system and the percentage tissue water content calculated. Colonic tissue samples were frozen to assay for myeloperoxidase activity for the ZnSA groups. Groups of twelve mice were used.

### The effect of oral administration of ZnSA, CuSA and zinc chloride on formalin induced colonic inflammation

Male BK:A mice (20–25 g). in treatment groups received either ZnSA, CuSA or zinc chloride (1.0 and 5.0 mmol kg^−1^) orally. ZnSA and CuSA were suspended in 0.2 mL of 0.9% saline whereas zinc chloride was dissolved in distilled water. A control group received 0.2 mL of 0.9% saline for ZnSA and CuSA and 0.2 mL of distilled water for the zinc chloride group. Drug or vehicle was administered twice daily 3 days prior to, on the day of and 1 day after formalin challenge. Colonic inflammation was induced via the local instillation of 0.2 mL of 2.0% formalin using a trocar needle. Insertion was 2 cm proximal to the anus. A control group received 0.2 mL of 0.9% saline intrarectally. All animals were killed 2 days after formalin challenge. Colons were dissected and examined for signs of inflammation using a graded scoring system and the percentage tissue water content calculated. Colonic tissue samples were frozen to assay for myeloperoxidase activity for the ZnSA group. Groups of six mice were used for the studies on ZnSA and CuSA and seven mice for the study using zinc chloride.

### Trinitrobenzenesulfonic acid (TNB) induced colitis in Rats (Morris et al. 1984, 1989)

#### Dosage

Under light anaesthesia, male Wistar rats (200–250 g) received 0.25 mL of solution intrarectally using a trocar needle. Insertion was around 7 cm proximal to the anus. An ethanol control group received 0.25 mL of 50% ethanol. Test animals received, 0.25 mL of 60 mg kg^−1^ TNB solution, 0.50 mL of 60 mg kg^−1^ TNB solution and 0.25 mL of an 120 mg kg^−1^ TNB solution in 50% ethanol respectively. A second study group received 0.25 mL of 80 mg kg^−1^ TNB solution or 0.25 mL of an 120 mg kg^−1^ TNB solution dissolved in 50% ethanol using a modified trocar needle (vide infra). An ethanol control group received 0.25 mL of 50% ethanol using the modified needle. Animals were killed at the stipulated time and examined for signs of inflammation using the scoring system.

#### Time course

Under light anaesthesia, male Wistar rats (200–250 g) received 0.25 mL of solution intrarectally using a modified trocar needle. Insertion was around 7 cm proximal to the anus. Test animals received 0.25 mL of an 80 mg kg^−1^ TNB solution in 50% ethanol. Control animals received 0.25 mL of 50% ethanol. The time points studied were 12, 24 h and 3, 5 and 7 days. Animals were killed a week later and examined for signs of inflammation using the scoring system (vide infra).

#### Modifications to the trocar needle

A length of narrow plastic tubing was inserted over a 4 cm long trocar needle to produce a final length of 7 cm. The end of the tubing was sealed and holes were made down the sides of the tubing; one hole either side, 4 mm apart to a length of 2.4 cm from the tip. This ensured good penetration and good fluid distribution.

### The effect of oral administration of SASP, ZnSA and sodium 5-(p-sulfophenylazo-)-salicylidenealanine-zinc (AzZnSA) on TNB induced colonic inflammation

Colitis was induced in male Wistar rats (180–220 g) under light anaesthesia via the rectal instillation of 0.25 mL of an 80 mg kg^−1^ solution of TNB dissolved in 50% ethanol using a modified trocar needle. Instillation was 7 cm proximal to the anus. One control group received 0.25 mL of 50% ethanol intrarectally, another control group received 0.25 mL of 0.9% saline intrarectally. The following experiments were not conducted blind. Male Wistar rats (180–220 g) in drug treatment groups received sulfasalazine, ZnSA or AzZnSA orally (1.5 × 10^−4^, 3.0 × 10^−4^, 7.5 × 10^−4^ mol kg^−1^ per day) in 1% hydroxyethylcellulose. A control group received 0.5 mL of 1% hydroxyethylcellulose. Drug or drug vehicle was administered one day prior to, on the day of and daily following the induction of colitis. Five rats were used in this study. The lowest dose of SASP used here is based on the clinically used dosage (60 mg kg^−1^ eq for rats). Animals were killed 7 days after TNB instillation. Colons were dissected out and examined for signs of inflammation using the graded scoring system. Colonic tissue samples were frozen to assay for myeloperoxidase activity.

### Scoring system

For studies using the formalin induced colitis a modified scoring system base on that reported by Morris et al. (TNB model) was employed (Morris et al. 1984, 1989) where

0 No signs of mucosal inflammation,
1 Erythema,
2 Any two of erythema, oedema or diarrhoea,
3 Any three of erythema, oedema, diarrhoea, pin head ulceration or stricture.
4 All of the above signs plus pronounced or clearly defined ulceration.
5 As 3 plus severe, extensive ulceration and/or severe structural damage.

### Measurement of oedema

A sample of colonic tissue (2 cm) was removed and weighed when wet. This is dried on a balance until it reaches constant weight (typically 45 min), from which the dry weight and percentage water is calculated.

Myeloperoxidase assay: MPO activity was conducted using the modified method of Bradley et al. 1982). 2 cm of colon was finely divided in 1 mL of 0.5% hexadecyltrimethyl-ammonium bromide (HTAB) in 50 mM K_3_PO_4_ buffer. The tissue was transferred to a homogenising tube taking care to transfer all materials (1 mL HTAB solution rinse was applied) and the mixture homogenised on ice. The mixture was transferred to assay tubes. All containers were rinsed with a further 1 mL of HTAB solution to give a final volume of 3 mL. The sample was sonicated for 10 s in an ice bath and then subjected to a three freeze thaw cycles in a dry ice bath. The sample was re-sonicated and then centrifuged. 1 mL of the supernatant was added to 2.9 mL of 50 mM K_3_PO_4_ buffer (pH 6) containing 0.167 mg mL^−1^ of o-dianisidine di-hydrochloride and 0.0005% hydrogen peroxide. The reaction was stopped after 5 min by the addition of 0.1 mL of 1.2 M hydrochloric acid. The absorbance of the resulting solution was measured at 400 nm.

Myeloperoxidase activity and percentage tissue water values were expressed as the mean ± standard error of the mean. Colitic scores were expressed as median and range. In the figures (vide infra), a large error bar usually indicates one value outwith the population around the median. Group comparisons were done using the Mann–Whitney *U* test. Where more than two groups were involved the results were analysed for difference using one-way analysis of variance and the significant differences between groups were examined by the Newman-Keuls range test at 5% level of significance. Asterisks denote significance to colitic controls unless otherwise stated.

## Results and discussion

### Synthesis and structure

**Fig. 2:**
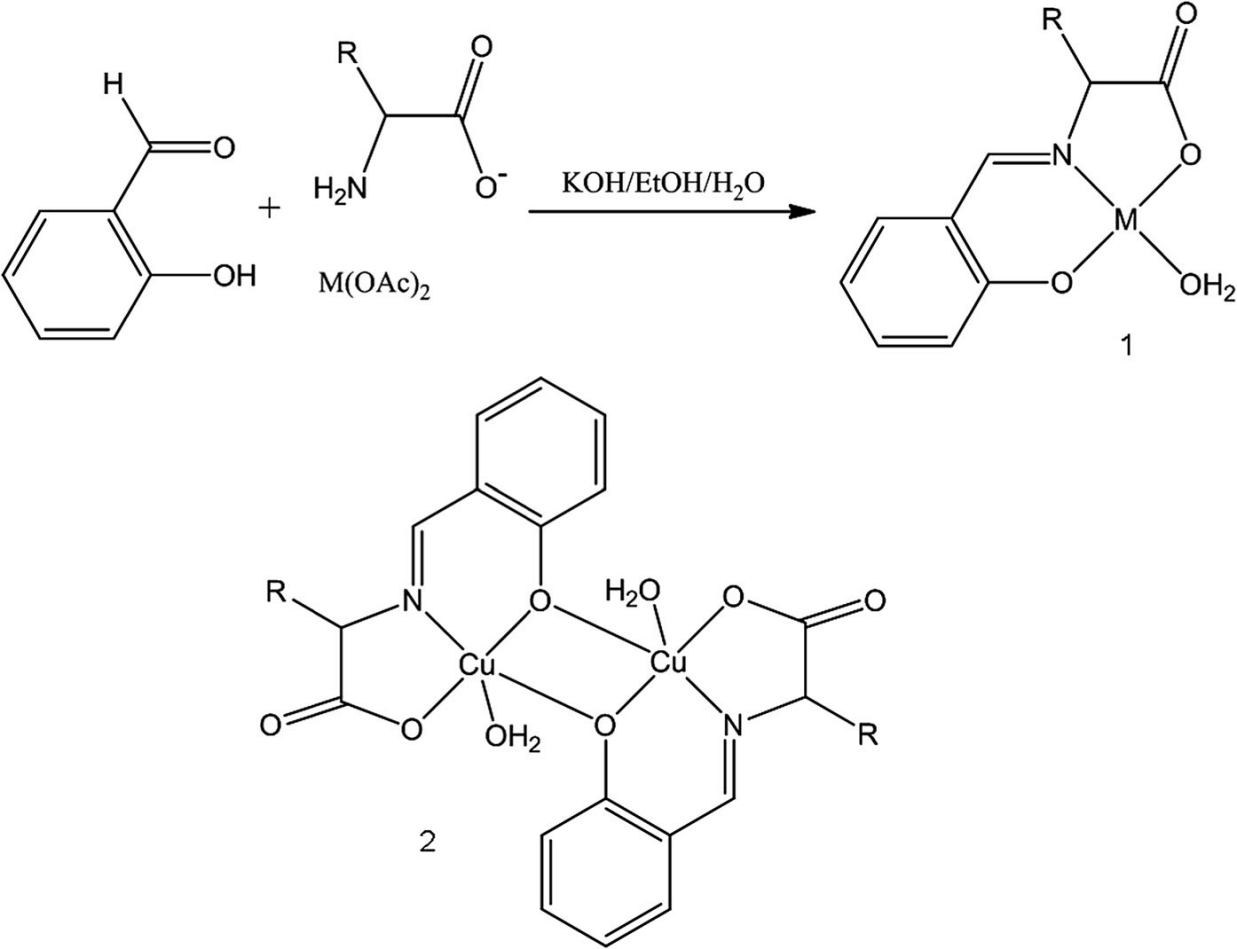
The synthetic scheme. The products form in most cases as monomers (*1*) or oligomers (Ueki et al. 1969; Warda 1998; Garcia-Raso et al. 2000; Shova et al. 2000; Li et al. 2007). Oligomerisation occurs via bridging carboxylate groups. CuSA and CuSG have crystallographically characterised dimeric forms (Li et al. 2007; Warda 1998)

A large number of Schiff base complexes of zinc and copper have now been reported. These are formed by the condensation of an aldehyde and amine in the presence of the desired metal cation, typically as the acetate. Although we wished to maintain a therapeutic link with 5-ASA (Fig. 2), we did not wish to use the amine functionality on 5-ASA as the basis of the Schiff’s base linkage as we envisaged using it later in the further development of the system to incorporate further active organic moieties (vide infra). We thus turned our initial attention to the carboxylate group as a primary site for chemical modification, but in its reduced form i.e. the aldehyde, as this not only provides us with a suitable commercially available aldehyde i.e. salicylaldehyde (Fig. 2) it introduces a hydrolytically unstable bond into the complex. A hydrolysis reaction occurring in vivo would allow the complex to degrade and slowly release the active agents brought together in our formulation. A number of the salicylaldehyde based compounds chosen for development have been structurally characterised elsewhere (Ueki et al. 1969; Warda 1998; Garcia-Raso et al. 2000; Shova et al. 2000; Li et al. 2007). These demonstrate a range of structural forms depending on the nature of the co-crystalline entities. However, it is evident that all complexes of this type have a common core motif (1, Fig. 2), one which will persist in aqueous solution.

When constructing and testing poly-modal compounds it is preferable to build them sequentially. Thus the first stage is to construct complexes, which contain salicylaldehyde and either copper or zinc. To stabilise the motif we still require a non-active amine and we have elected to use simple amino acids in the initial survey. Although we were successful in preparing a number of simple Schiff base complexes of copper and zinc (Fig. 2), without the stabilising influence of the chelated metal the simple condensation products of the amino acid and salicylaldehyde were not stable in out hands. This observation mitigates against an investigation of the simple condensates as an independent control.

### Acid tolerance

**Fig. 3:**
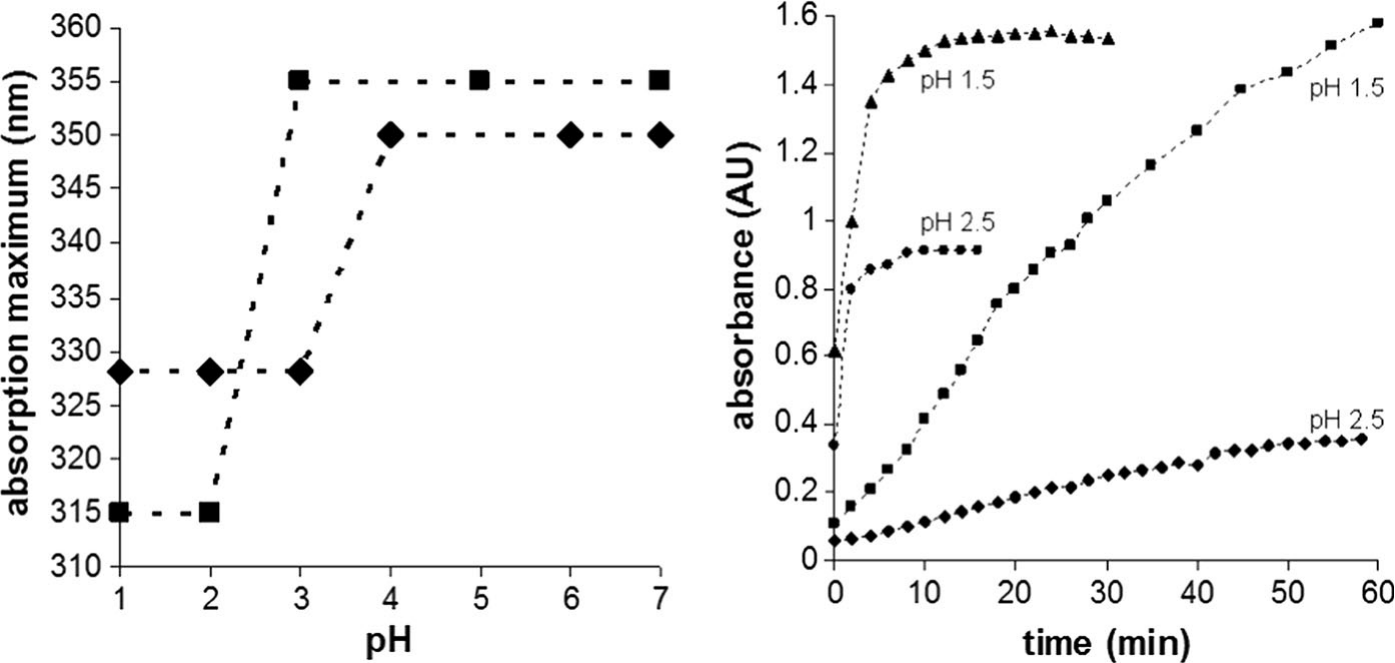
*Left* Spectrophotometric analysis of ZnSG (*black diamond*) and CuSA (*black square*) as a function of pH. ZnSA is moderately less acid tolerant. However, at pH’s >4 metal complex (λ_max_ 350) will persist in solution. At pH’s below 3 the complex hydrolyses to release salicylaldehyde (λ_max_ 325): *Right* The hydrolysis of ZnSA (*black diamond*) and CuSA (*black square*) in an octanol water mixture at pH 1.5 and 2.5 estimated as the increase in free salicylaldehyde (λ_max_ 325) in the octanol layer

Studies using PENTASA® have demonstrated that even when the release of the anti-colitic components is initiated in the stomach sufficient quantities of the active agent can still be achieved in the colon (Christensen et al. 1987). However, it would be preferable if significant amounts of the orally administered agent traverses the stomach and reaches the inflamed colon. Since the pH of the stomach can vary from 1.0 to 3.5 in the fed state rising to 4.0–5.0 in the unfed state, it is important to understand how these Schiff base complexes (Fig. 3) breakdown under different pH conditions (Gruber et al. 1987). This knowledge will make it possible to select putative compounds based on their predicted behaviour in the stomach.

A simple spectrophotometric analysis of the isolated metal complexes and salicylaldehyde demonstrate that the Schiff base complexes have an absorption maxima at 350 nm whereas the free salicylaldehyde has a maxima at 325 nm respectively. Thus by monitoring solutions of a given complex as a function of pH it is possible to show at which pH complex degradation is significant (Fig. 3). The study shows that both compounds will degrade at pH’s prevalent in the stomach. However, CuSA would seem to be more acid tolerant. This study does not provide any details on the rate of degradation. As such moving to an octanol water system allows for time dependant sampling. Subjecting the compounds to solutions at very low pH accelerates degradation to rates which can be observed by spectrophotometric means. From this study it can be seen that degradation of ZnSA is rapid (t_½_ ~ mins) whereas CuSA is much longer lived (Fig. 3). This study suggests that the copper complex has a longer lifetime and is better placed to traverse the hostile environment either as a suspended material or adsorbed onto larger moieties. It is notable that the choice of metal centre can have a profound effect on the degradation process.

### The effect of ZnSA and CuSA on Formalin induced colonic inflammation

**Fig. 4:**
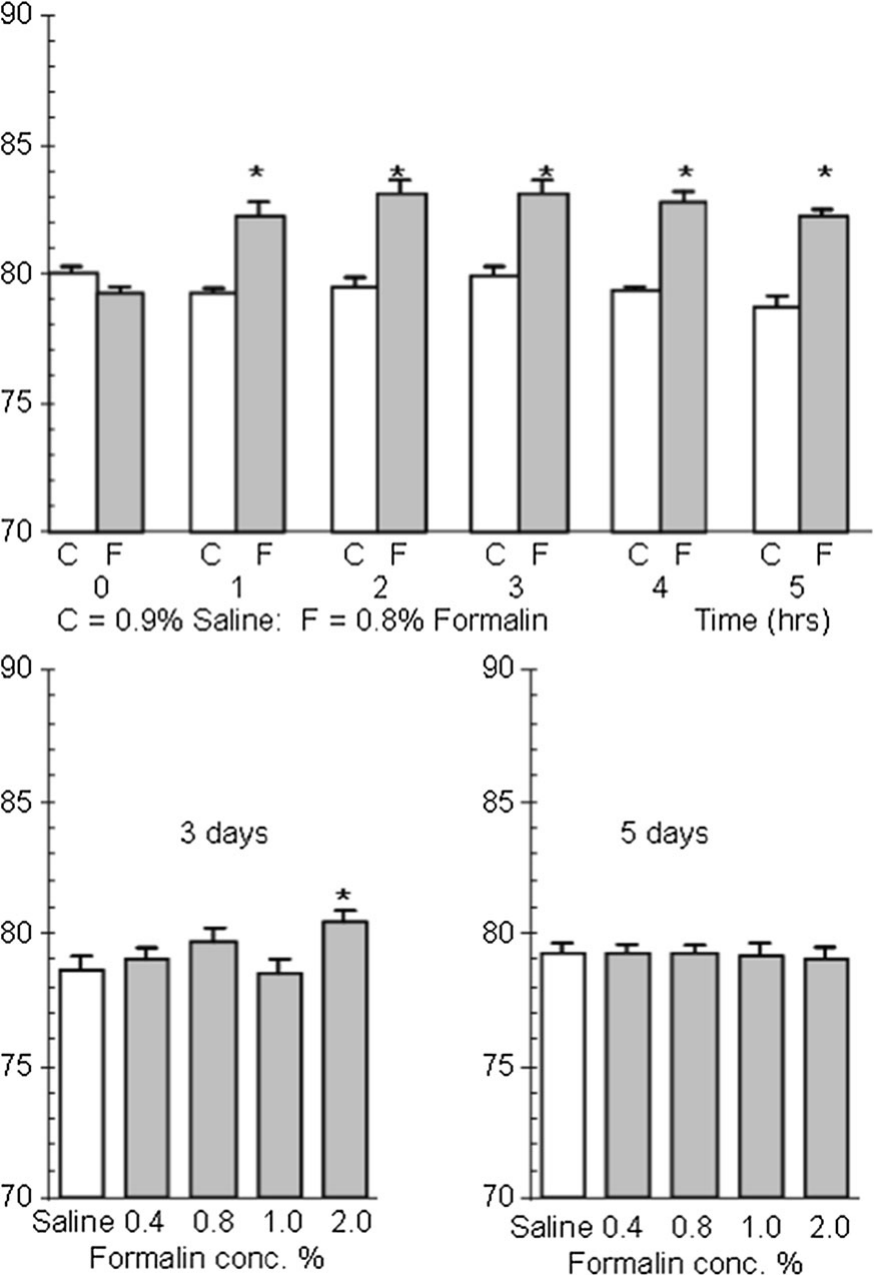
*Top* the effect of intracolonic administration of 0.8% formalin (solid column; n = 10) on percentage tissue water content during a period of five hours. Saline (0.9%) control (open column n = 6). Results are presented as mean ± SEM. Asterisks denote significant difference from saline control (*p* < 0.05; Mann–Whitney *U* test). *Bottom* the effect of intracolonic administration of formalin (0.0–2.0%) on percentage tissue water content at 3 and 5 days during a period of 5 h. n = 6 for all groups. *Asterisks* denote significant differences between groups (*p* < 0.05); one way analysis of variance, Neuman–Keuls range test

Formalin is one of the components of immune complex mediated colitis and is capable of initiating an inflammatory response in its own right. This observation made the formalin induced colonic inflammation (FICF) model particularly appropriate for the needs of this study (Walsh et al. 1987). However, prior to be being challenged with any of the potential anticolitic compounds we required to optimise the model to identify an appropriate protocol for the induction of colonic inflammation. BK:A mice received an intrarectal instillation of formalin (0.0, 0.4, 0.8, 1.0 and 2.0%) in two groups. The first group were sacrificed after 3 days and the second group after 5 days after which the colonic tissues from both groups were examined for changes in their colitic score (Fig. 4). The study indicates that a suitable inflammatory response was present at 3 days but that the response had dissipated at day 5. Macroscopic features associated with inflammation were observed during this test at day 3 i.e. oedema (incidence 4/6), mucus (3/6) and ulceration (1/6). Although the lower concentrations of formalin had no effect on tissue water content at day 3 or 5, animals receiving 0.8% formalin did show a median colitic score of 1 at day 3 indicating that the tissue showed either erythema, excess mucus or oedema. A second validation study was conducted (Fig. 4) using a shorter time period (0–5 h) but with a fixed concentration of formalin (0.8%). This shows that the inflammatory response initiated by formalin presents very quickly in the model. A model in which the induction time for inflammation is short (~2 h) and can last days is particularly appropriate to the needs of this study.

**Fig. 5:**
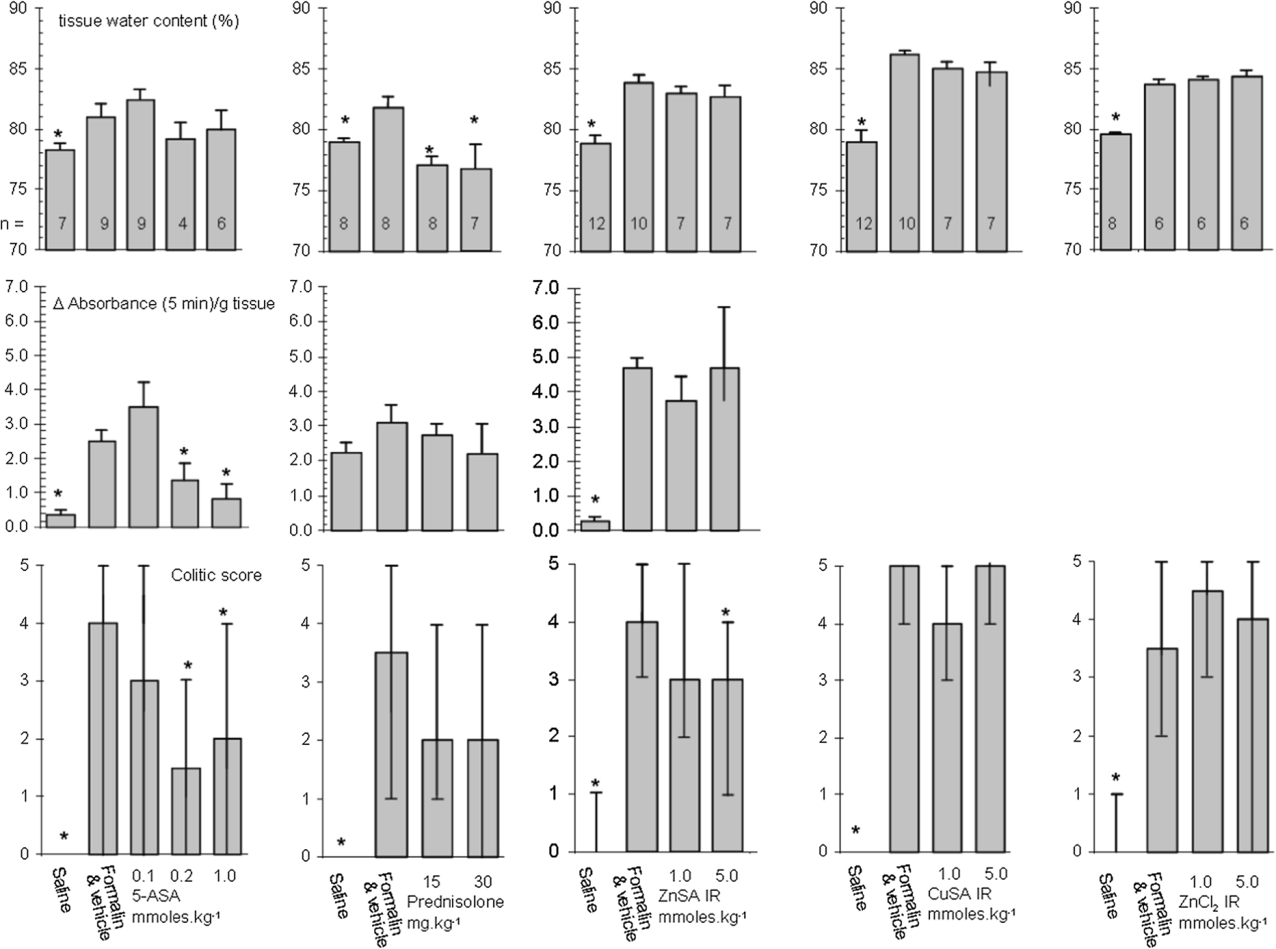
The effect of intrarectal 5-ASA, prednisolone i.p., intrarectal ZnSA, CuSA and ZnCl_2_ on percentage tissue water content (*top*), myeloperoxidase activity (*middle*) and colitic score (*bottom*) 2 days after intrarectal formalin (2%) challenge. Drug was administered 3 days prior to, on the day of and 1 day after formalin challenge. Groups studied were saline control, formalin and vehicle and compound. The number in each group is identified on the data associated with tissue water content. Results are presented as mean ± SEM. for tissue water content and myeloperoxidase activity and as median and range for the colitic score. Asterisks denote significant difference to control (*p* < 0.05); one way analysis of variance, Neuman–Keuls range test. The poor performance of prednisolone in this study is ascribed to the different method of administration and how this modulates the dosage applied

**Fig. 6:**
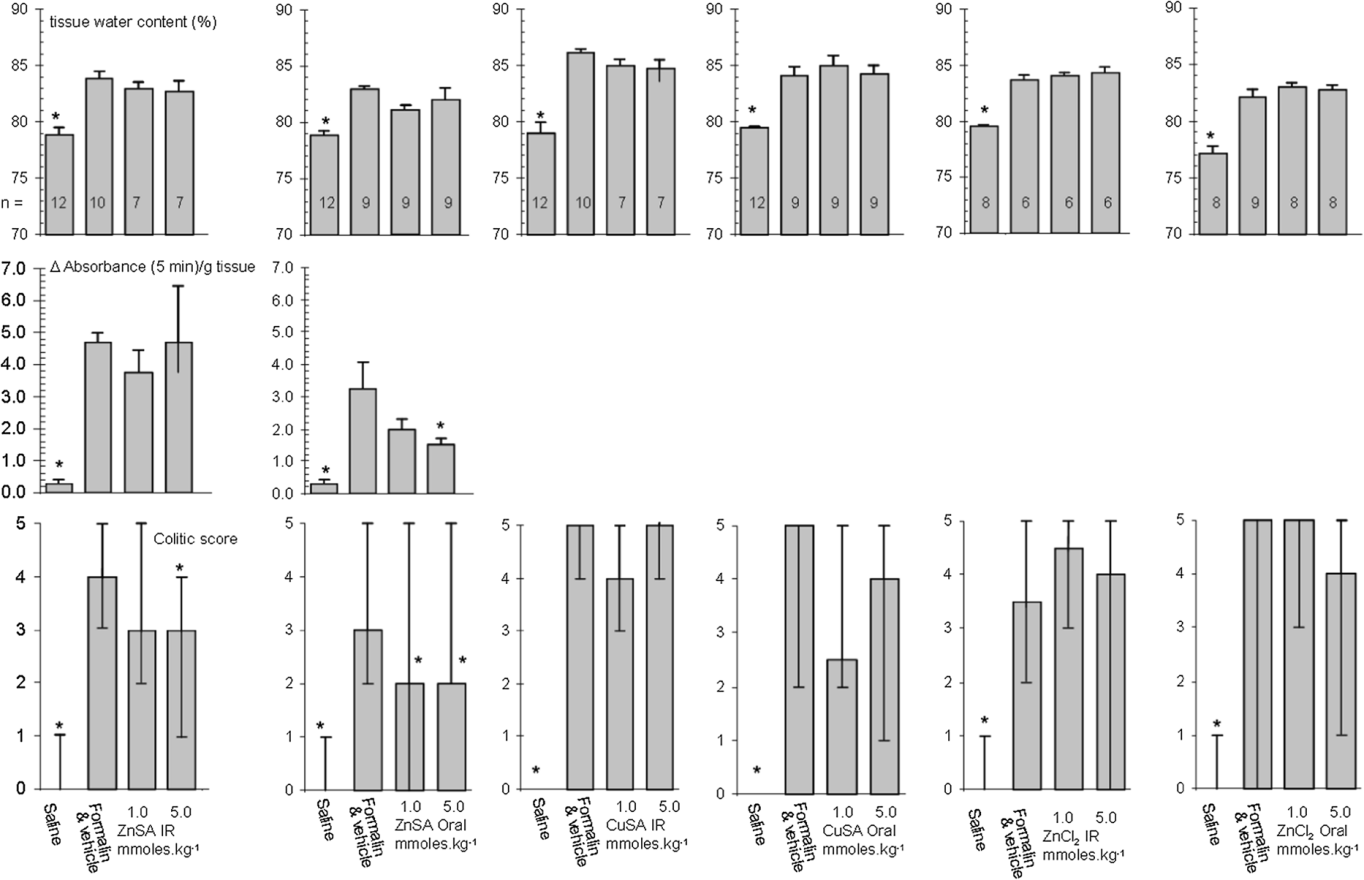
The comparison of intrarectal and oral administration of ZnSA, CuSA and ZnCl_2_ on percentage tissue water content (*top*), myeloperoxidase activity (*middle*) and colitic score (*bottom*) 2 days after intrarectal formalin (2%) challenge. Drug was administered 3 days prior to, on the day of and 1 day after formalin challenge. Groups studies were saline control, formalin and vehicle and compound. The number in each group is identified on the data associated with tissue water content. Results are presented as mean ± SEM. for tissue water content and myeloperoxidase activity and as median and range for the colitic score.* Asterisks* denote significant difference to control (*p* < 0.05); one way analysis of variance, Neuman–Keuls range test

We wished to model a simple treatment regimen which modelled therapeutic practice and as such decided to administered drug or vehicle 3 days prior to, on the day of, and 1 day after formalin challenge. Using the data above (Fig. 4) we opted to employ the higher concentration of formalin (2.0%) over a 2 day period. These compromises would allow inflammation to be established quickly and be sustained during the treatment period. The anti-colitic properties of ZnSA and CuSA were thus tested using the FICF model. The study was controlled using 5-ASA, prednisolone and zinc chloride (ZnCl_2_). 5-ASA, ZnSA, CuS and ZnCl_2_ at equimolar amounts were administered intrarectally whereas prednisolone was administered by i.p. injection. Samples retrieved from the colons were used to calculate the tissue water content, myeloperoxidase assay and colitic score (Fig. 5). The myeloperoxidase assay did not perform in an acceptable manner in the presence of CuSA and consequently the data was not recorded for this compound. Prednisolone significantly (*p* < 0.05) lowers the tissue water content of colonic tissue compared to formalin. In contrast only 5-ASA has a significant effect (*p* < 0.05) on myeloperoxidase activity. However, the analysis of the overall anti-colitic behaviour of the compounds estimated using the scoring system not only confirms the efficacy of 5-ASA but supports the conclusion that high dose ZnSA significantly reduces (*p* < 0.05) the median colitic score compared to that of the formalin treated group. In contrast the corresponding copper species had little effect on any of the parameters measured. The pH studies discussed above (Fig. 3) show that ZnSA has the more rapid degradation profile which suggests that significant amounts of simple zinc salts might be being generated in the colitic zone during analysis. Testing this hypothesis by the application of ZnCl_2_ instead of ZnSA (Fig. 5) clearly shows that zinc alone is having little effect. It is evident that it is the organic component or metal complex which is giving rise to the positive anticolitic result. The study was reformulated and the compounds administered orally (Fig. 6). The 5-ASA and prednisolone controls were not included at this juncture as we were particularly interested in the direct comparison of the administration route of the two compounds. Consistent with the above CuSA still shows little evidence of any anticolitic behaviour by oral administration. However, ZnSA demonstrated improves anticolitic properties when administered orally. Not only did ZnSA show a significant effect through its impact on the median colitic score, there is now evidence that it is having a beneficial effect on myeloperoxidase activity at higher dosages. Once again we could not identify any anti-colitic properties for ZnCl_2_ alone. Thus despite the poor stability profile of ZnSA (Fig. 3) it would seem that a portion of the compound is reaching the colon intact where it has a beneficial effect on the inflammatory process. Although key data points do indicate that ZnSA is a good lead compound at no point do we see any evidence for a dose related response at this juncture.

It should be noted that the compounds (ZnSA, CuSA) being tested above are bimodal i.e. they release zinc/copper and a 5-ASA precursor into the region of colonic insult. Based on the data it was decided to abandon copper as a potential anti-colitic moiety and focus our attention on zinc. The design and synthesis of ZnSA (Fig. 2) employed alanine as a redundant amine for Schiff base formation. However, the methodology which allows the alanine moiety to be replaced by a biologically active amine is well established. Indeed the synthesis of the penicillamine and cysteine adducts have been reported (MacDonald et al. 1979, 1982; Chohan et al. 2007). Thus of greater interest and potentially greater impact is to strengthen the link between 5-ASA and our salicylidene moiety (Fig. 2) by modifying the salicylaldehyde moiety.

### Trimodal complexes

**Fig. 7:**
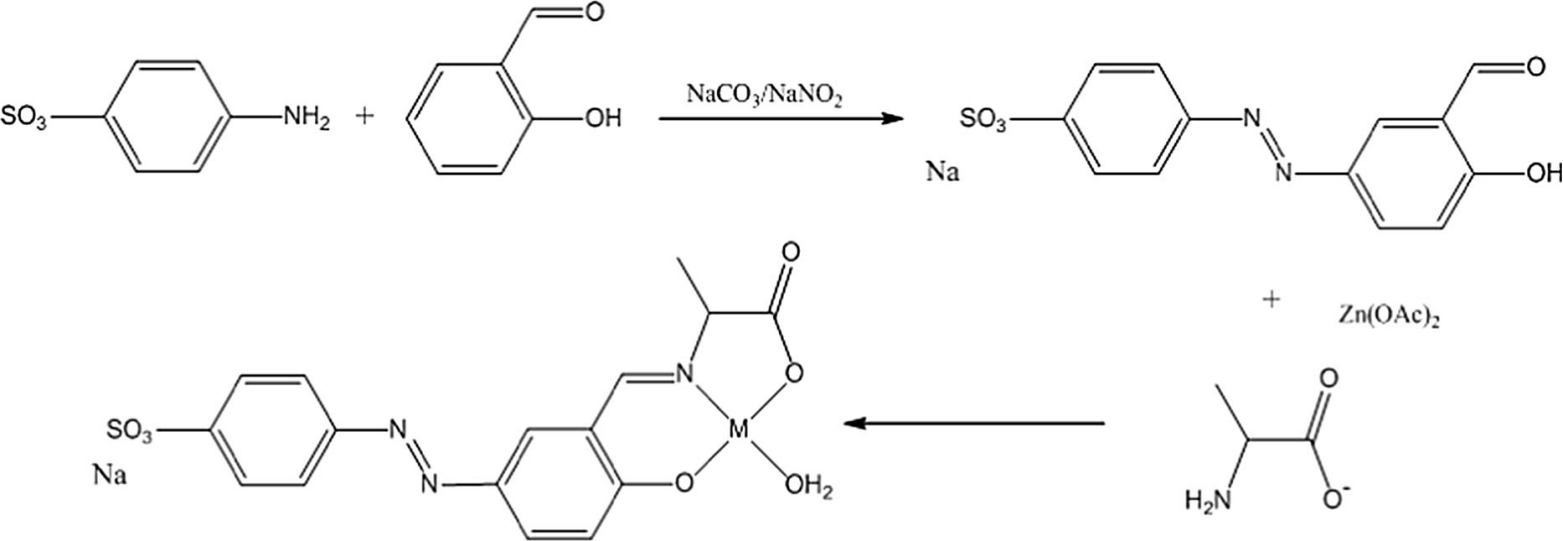
The synthesis of the trimodal zinc complex AzZnSA. The compound now incorporates zinc, a 5-ASA precursor and an azo entity which models the active moiety in SASP

The desire to focus on further modification to the salicylaldehyde ring is driven by the possibility of introducing an azo-linkage into the compound. Electronic effects will drive this modification to the 5 position on the aryl ring (Fig. 7) and as such post modify the salicylaldehyde into a more apposite 5-ASA analogue. However the introduction of an azo group introduced at this position means that we will have effectively stabilised a Schiff base complex of a sulfasalazine analogue (Fig. 7).

**Fig. 8:**
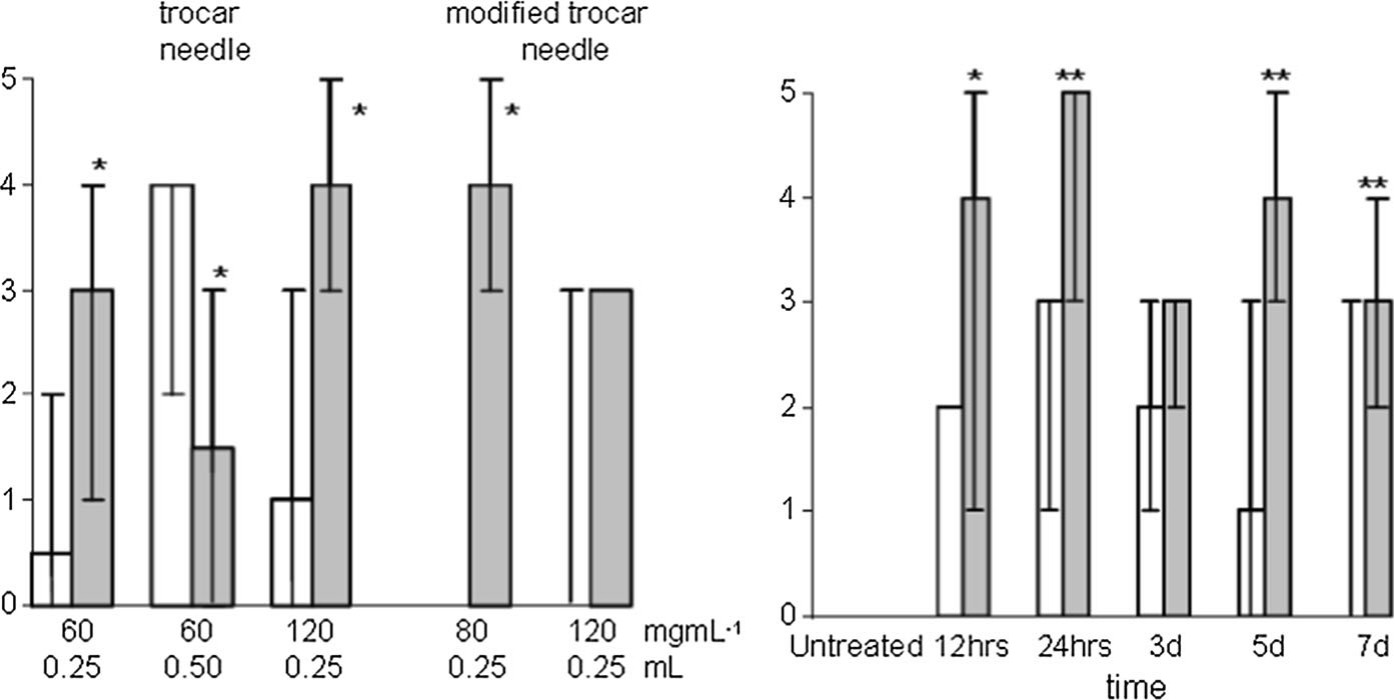
*Left* the effect of intracolonic administration of 50% ethanol (*open columns*) and TNB in 50% ethanol at various concentrations and volumes (*solid column*) on colitic score. Results are represented as median and range. *Right* the time course of intrarectally administered ethanol (50% *open columns*) and TNB (80 mg mL^−1^ in 50% ethanol) on colitic score. n = 5 for all groups. *Asterisks* denote significant differences between groups (*p < 0.05, **p < 0.01); one way analysis of variance, Neuman–Keuls range test

The studies above using BK:A mice confirmed that the zinc compounds displayed anticolitic activity and furthermore that they were active via the oral route. However, modelling colitis is extremely difficult and care must be exercised not to rely too heavily on favourable results from a single model. As such it was decided to introduce a second animal model for the testing of the second generation compounds, moving to the trinitrobenzenesulphonic acid (TNB) mediated colitic model using larger Wistar rats (Morris et al. 1984, 1989). Moving to a larger animal had the added advantage that there would be more material and thus there would be less variation within the data. Since the trimodal compound being tested here has greater similarity to sulfasalazine (SASP) we also replaced 5-ASA with SASP as the control in these tests. However, prior to be being challenged with any of the anticolitic compounds we are again required to optimise the model to identify an appropriate protocol for the induction of colonic inflammation. Wistar rats received an intrarectal instillation of TNB at a variety of concentrations and volumes (Fig. 8). At low dosages (60 mg mL^−1^) we observed that the incidence and rate of ulceration were inconsistent. At higher concentrations (120 mg mL^−1^) the mortality rate was too high. This was thought due to the extremely localised introduction of the TNB solution to the colon. Consequently a modified needle was employed which allowed the solution to be more evenly distributed within the colon during TNB administration. The application of TNB using the modified needle gave agreeable results at 80 mg mL^−1^ TNB, however, the mortality rate at 120 mg mL^−1^ was still inferior and as such we elected to go forward with a dose regimen of 80 mg mL^−1^. The time course supports the view that 80 mg mL^−1^ is an appropriate concentration. All the animals (2 × 6 × 6, Fig. 8) survived to day seven. The data at 5 and 7 days indicated that the inflammatory model has a long duration. Consequently, we elected to employ a 1 week gestation period.

**Fig. 9:**
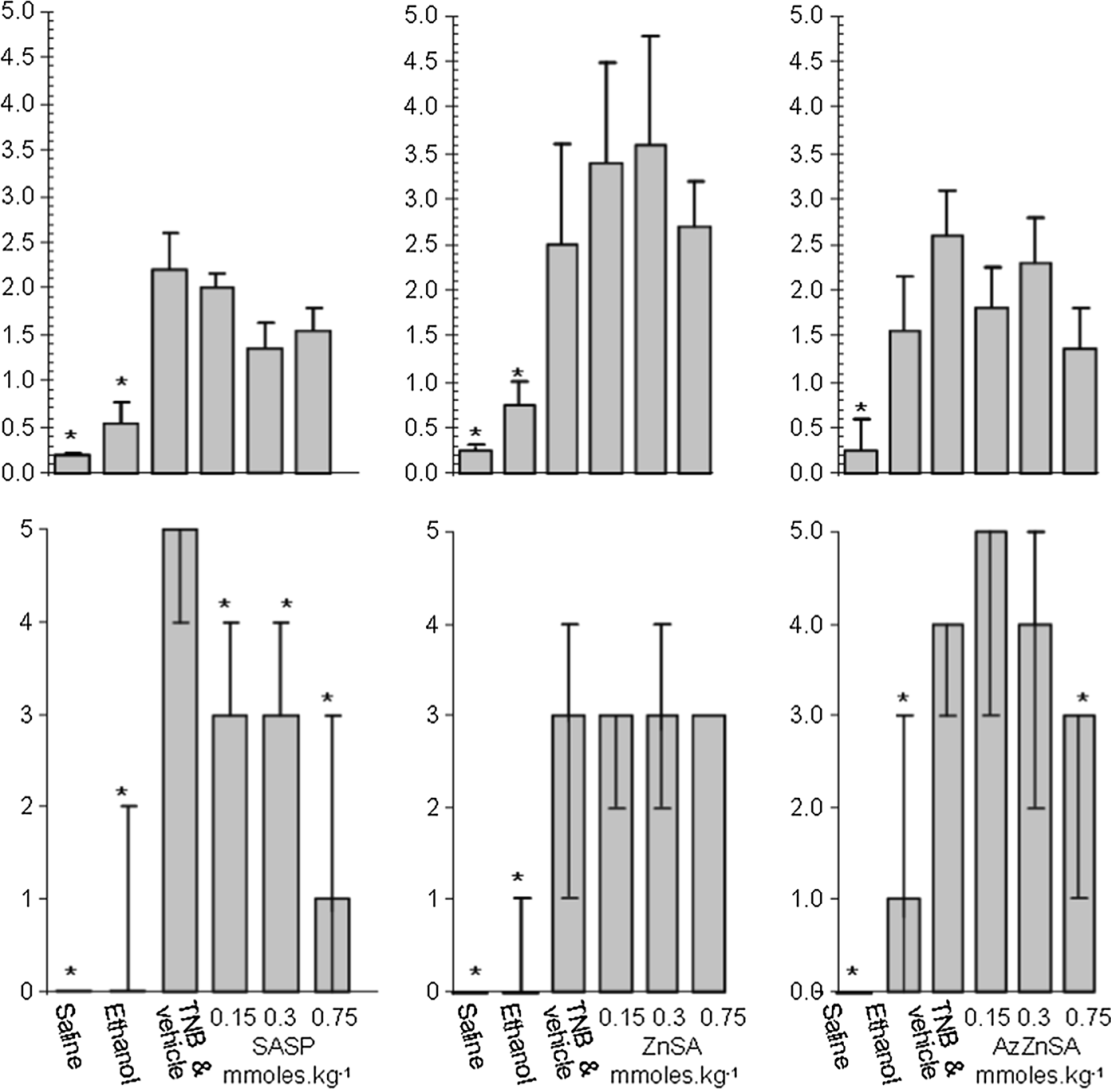
*Left* the effect of oral SASP, ZnSA and AzZnSA (1.5 × 10^−4^, 3.0 × 10^−4^ and 7.5 × 10^−4^ mol kg^−1^) on myeloperoxidase activity (*top*) and colitic score (*bottom*) 7 days after induction of colonic inflammation with TNB (0.25 mL 80 mg kg^−1^ in 50% ethanol). n = 5. Drug was administered one day prior to, on the day of and daily after induction of colitis. Groups studies were saline control, ethanol control, TNB and vehicle, SASP, formalin and vehicle and compound. Results are presented as mean ± SEM. for myeloperoxidase activity and as median and range for the colitic score. *Asterisks* denote significant difference to control (*p* < 0.05); one way analysis of variance, Neuman–Keuls range test. The median colitic score (3) of the TNB vehicle group in the ZnSA study is low

**Fig. 10:**
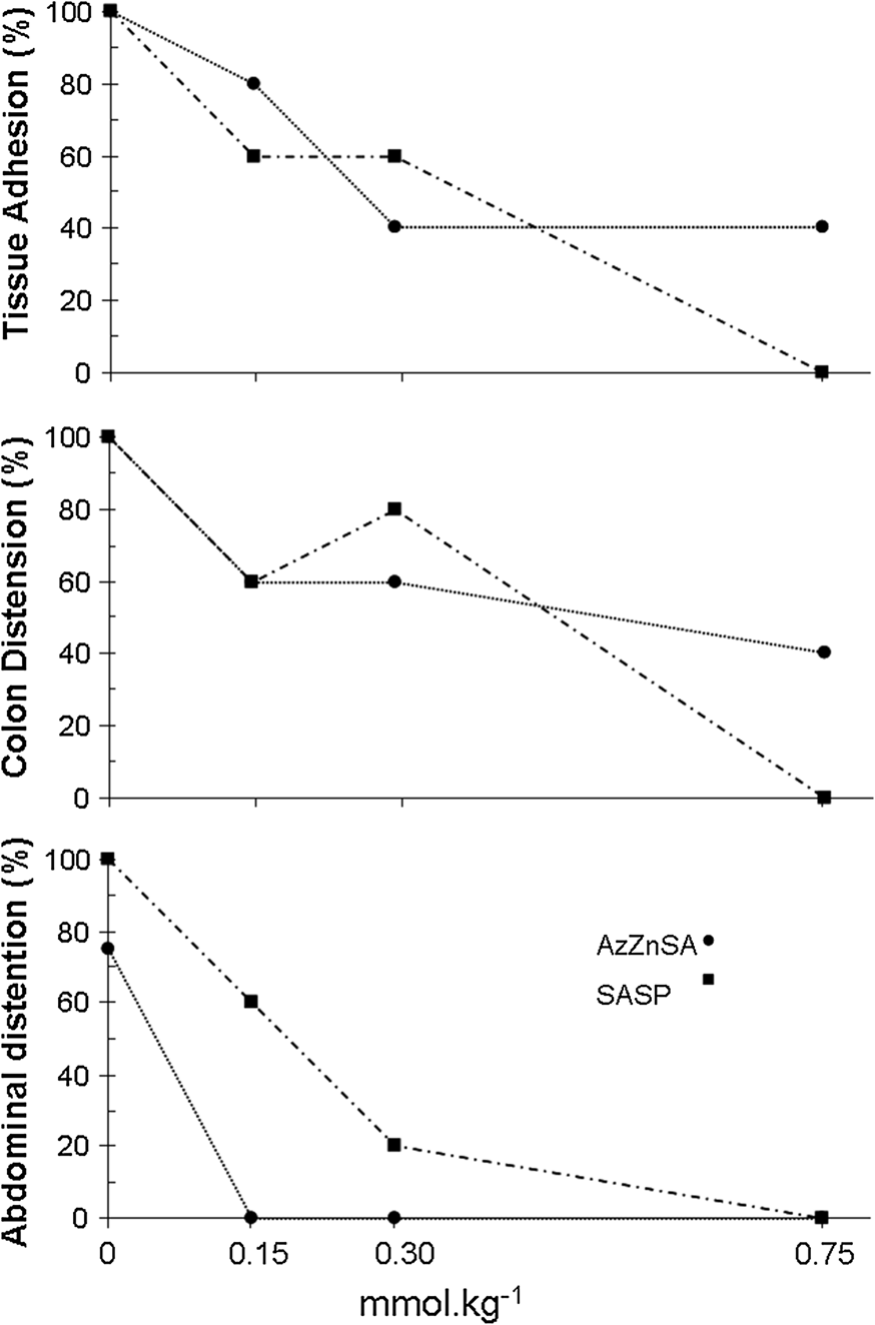
The dose relationship of AzZnSA and SASP on the incidence of tissue adhesion, colon distension and abdominal distension in TNB induced colonic inflammation

The SASP treated group showed a significant increase in the colitic score compared to the TNB/drug vehicle group (Fig. 9) with the median scores falling in line with the dosage. This is confirmed by a more in-depth analysis of incidence of tissue adhesion, colon distension and abdominal distension which indicated that SASP affords symptomatic control of TNB-mediated colitis (Fig. 10). Myeloperoxidase activity in all groups receiving TNB was significantly higher than the saline and ethanol control groups. However, there was no significant reduction in myeloperoxidase activity between the TNB drug vehicle group and the drug treated group. Thus the reduction in colitic score produced by SASP is not paralleled by a reduction in neutrophil activity. This is consistent with the observation that treatment with 5-ASA (100 mg kg^−1^) produces significant reductions in colonic damage, colon wet weight and the incidence of adhesion but also did not affect colonic myeloperoxidase activity or LTB_4_ synthesis (Wallace 1988).

Treatment with ZnSA orally did not produce a significant reduction in colitic score or reduction in myeloperoxidase activity (Fig. 9) in the TNB induced colonic inflammation model. This observation contrast markedly with the data obtained earlier in the mouse model (Fig. 6). In the formalin induced mouse model neutrophil flow was rapid and of short duration and is thus is probably a good marker for the determination of the inflammatory response. In the TNB model, although neutrophil influx is rapid it is maintained for 7 days (Fig. 8). This suggests that a potent anti-inflammatory would be required to inhibit myeloperoxidase activity in the TNB model. In contrast to ZnSA, AzZnSA produced a significant reduction in the median colitic score compared to the median values for the TNB/drug vehicle group (Fig. 9). The group treated with the highest dosage of AzZnSA demonstrated an absence of tissue adhesion, colonic distension and abdominal dissention compared to the TNB group (Fig. 10). However, similar to ZnSA, AzZnSA was not effective in reducing myeloperoxidase activity. It can be concluded that AzZnSA was effective in reducing the symptoms of colitis but not neutrophil activity. This observation is similar to that observed for SASP in the same model. Plotting the individual scores for tissue adhesion, colonic distension and abdominal distension in the SASP and AzZnSA treatment groups suggest there is a similar dose related profile for the two species. It can be concluded that the diazotisation of the Schiff base complex ZnSA has produced a more potent compound and one comparable to SASP. The reasons for this are probably threefold. The azo group is assisting in the delivery of the complex to the colon (c.f SASP), the diazotisation reaction has chemically altered the salicylaldehyde into a reduced form of 5-ASA and finally the incorporation of sulfanilic acid, similar to a sulfapyridine, may be acting to prevent injury (Cho et al. 1987).

## Concluding remarks

The treatment of ulcerative colitis is limited to two main approaches: corticosteroid therapy and SASP therapy. In the case of resistance to these azathioprine is administered. Thus there is still a need for new approaches to the treatment of this condition. The desire to have orally administered drugs requires the synthesis of species which can survive the pH conditions of the stomach. We have approached this problem here by designing Schiff base species which serve to protect the active agent during transit. We focussed our attention here on copper and zinc. While we have observed that zinc performs better than copper more effort is required to screen alternative metal centres which can stabilise Schiff base linkages.

We have been able to show that orally administered ZnSA at 1.0 × 10^−3^ mol kg^−1^ significantly reduces the colitic score (60%) and myeloperoxidase activity (58%) in an acute colitis model (mouse). However, sadly this compound does not provide any significant protection in a more chronic (TNB induced colitic) model. It is possible that ZnSA only functions in acute colitis. Diazotisation of the salicylaldehyde generates a better 5-ASA analogue and a compound which also better models SASP. This species is observed to provide anticolitic properties in the chronic TNB induced colitis model commensurate with SASP. Significantly, the trimodal compound designed here while showing great promise can be modified much further and there remains great scope for further development. For example, this study has relied exclusively on simple non bio-active amino acids such as alanine to furnish the Schiff base linkages. This moiety could easily be replaced by more active amino acids species such as cysteine and penicillamine which have antioxidant properties in their own right. Alternatively, the incorporation of species such as lipoamide might allow the compounds to be specifically targeted to the gut microfloral as a release mechanism. Finally the sulfanilic acid used in the diazotisation reactions could itself be replaced by more bioactive aryl species. Alternatively the analino function on the sulfanilic acid can itself be used to carry a further bioactive entity (c.f. SASP). If any of these modification can be shown to enhance the anti-colitic properties of the motif reported here, then the compound generated could potentially be able to outperform the anti-colitic properties of SASP.
